# Methylation and Loss of Secreted Frizzled-Related Protein 3 Enhances Melanoma Cell Migration and Invasion

**DOI:** 10.1371/journal.pone.0018674

**Published:** 2011-04-08

**Authors:** Elin J. Ekström, Victoria Sherwood, Tommy Andersson

**Affiliations:** Cell and Experimental Pathology, Department of Laboratory Medicine, Lund University, Clinical Research Centre, Skåne University Hospital, Malmö, Sweden; University of Birmingham, United Kingdom

## Abstract

**Background:**

Wnt signaling is important in development and can also contribute to the initiation and progression of cancer. The Secreted Frizzled Related Proteins (SFRPs) constitute a family of Wnt modulators, crucial for controlling Wnt signaling. Here we investigate the expression and role of SFRP3 in melanoma.

**Methodology/Principal Findings:**

We show that SFRP3 mRNA is down-regulated in malignant melanoma tumors as compared to normal/benign tissue. Furthermore, we found that SFRP3 expression was lost in the malignant melanoma cell lines, A2058, HTB63 and A375, but not in the non-transformed melanocyte cell line, Hermes 3A. Methylated CpG rich areas were detected in the SFRP3 gene in melanoma cell lines and their SFRP3 expression could be restored using the demethylating agent, 5′aza-deoxycytidine. Addition of recombinant SFRP3 to melanoma cells had no effect on viable cell numbers, but decreased cell migration and invasion. Wnt5a signaling has been shown to increase the migration and invasion of malignant melanoma cells, and high expression of Wnt5a in melanoma tumors has been connected to a poor prognosis. We found that recombinant SFRP3 could inhibit Wnt5a signaling, and that it inhibited melanoma cell migration and invasion in a Wnt5a-dependent manner.

**Conclusion/Significance:**

We conclude that SFRP3 functions as a melanoma migration and invasion suppressor by interfering with Wnt5a signaling.

## Introduction

Wnt signaling is essential in many different biological processes during development and tissue maintenance. Tight homeostatic control of Wnt signaling is crucial for the organism, as aberrant Wnt signaling can lead to developmental defects and plays important roles in many cancers [1]. The Frizzled proteins are a family of G-protein coupled receptors considered to be the principal receptors for Wnt proteins. However, there are also other putative Wnt receptors such as Ror2 [2], [3]. All Wnt-ligands and most of their cognate receptors contain a cysteine-rich domain (CRD), through which their binding is thought to be mediated. Activation of Wnt signaling by proteins such as Wnt3a and Wnt1 activates canonical signaling that leads to inhibition of β-catenin proteolytic degradation. This results in β-catenin accumulation and subsequent transcription of β-catenin-dependent promoters via interaction with transcription factors such as TCF/Lef. In contrast, non-canonical Wnt signaling activated for example by Wnt5a and Wnt11, inhibits the transcriptional activation of β-catenin but also triggers calcium signaling and JNK activation [4], [5].

Control of Wnt signaling is executed by a variety of different modulators including the Dickkopf proteins, the Wnt Inhibitory Factor-1 and LRP5/6 Wnt co-receptors [1]. Wnt signaling is also regulated via the family of Secreted Frizzled-Related Proteins (SFRPs). There are five human SFRPs and all contain a CRD homologous to the Frizzled CRD, which binds to Wnt ligands [6]. SFRP proteins can inhibit activation of both canonical and non-canonical Wnt signaling [7]. All SFRP members also share a conserved Netrin domain in common with tissue inhibitor of matrix metalloproteinases and Netrin-1 [8]. SFRPs are down-regulated in several cancers and this is often correlated with poor prognosis, as has been shown for breast, colorectal, and a number of other cancers [9].

Wnt signaling affects malignant melanoma progression [1], [4], [10]. Malignant melanoma comprises about 4% of skin cancers and 80% of skin cancer deaths are related to malignant melanoma due to its highly metastatic behavior. Once melanoma has spread there are currently few effective treatment options, thus there is a great need for a better understanding of melanoma progression [11]. Wnt signaling is crucial for cell fate determination of melanocytes from neural crest progenitor cells, and is connected to malignant melanoma development in several ways [10], [12]. Canonical Wnt signaling leads to differentiation and decreased proliferation of melanoma cells, and non-canonical signaling antagonizes this effect [13]. Non-canonical signaling also increases the migration and metastatic potential of melanoma cells [14], [15]. Increased expression of Wnt5a in malignant melanoma tumors is correlated with poor prognosis [16]. Conversely, canonical signaling leads to better prognosis in melanoma patients [13]. Currently the role of Wnt modulators in melanoma is poorly characterized.

Here, we show reduced SFRP3 expression in malignant melanoma tissues and cell lines compared to normal cells. We demonstrate that this down-regulation is related to methylation of the SFRP3 gene. Addition of SFRP3 to melanoma cells had no effect on the number of viable cells, but decreased migration and invasion in a Wnt5a-dependent manner. This work suggests that SFRP3 functions as a migration and invasion suppressor in malignant melanoma.

## Results

### SFRP3 mRNA is reduced in melanoma

**Figure 1:**
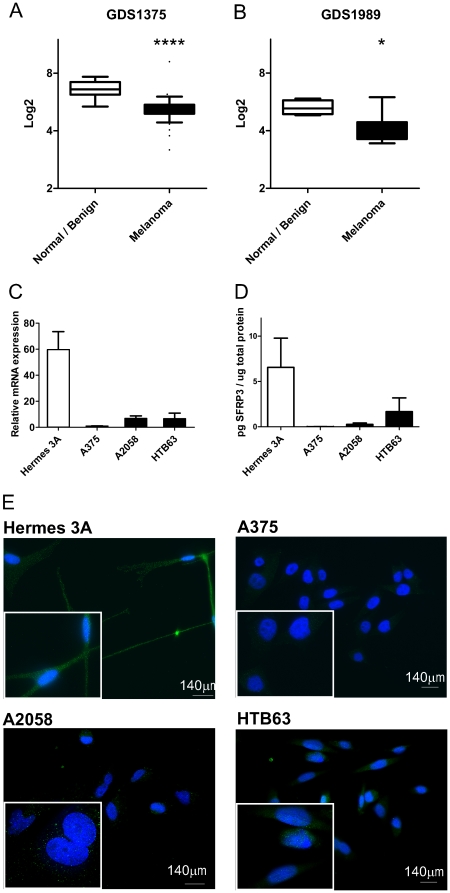
SFRP3 mRNA and protein levels are reduced in melanoma. *A*, Microarray data from Gene Expression Omnibus (GEO) dataset GDS1375, Normal/benign vs. Melanoma. *B*, Microarray data from GEO dataset GDS1989, Normal/benign vs. Melanoma. Bars represent the 95th and 5^th^ percentile. *C*, Basal SFRP3 mRNA expression in cell lines. A human non-transformed immortalized melanocyte cell line, Hermes 3A, and three malignant melanoma cell lines: A375, A2058 and HTB63 cells were analyzed for their SFRP3 mRNA expression using RT-QPCR. The data is representative of 3 independent experiments and the error bars represent standard deviation (SD) within one experiment. *D*, Basal SFRP3 protein levels in media from the indicated cell lines. The SFRP3 protein levels were normalized against total protein content. Error bars represent Standard Error of the Mean (SEM) (n = 4). Statistical analysis determined the following p-values; Hermes 3A vs. A375 p = 0,0025, Hermes 3A vs. A2058 p = 0,0051 and Hermes 3A vs HTB63 p = 0,1091. *E*, Basal SFRP3 protein expression in cell lines. Melanocytes and melanoma cells were analyzed for SFRP3 expression using immunofluorescence. Representative immunofluorescence images from 3 independent experiments. * = p<0.05, **** = p<0.0001.

The expression levels of SFRP3 mRNA were analyzed using two microarray datasets, GDS1375 [17] and GDS1989 [18], from the NCBI Gene Expression Omnibus database [19] (Figure 1A and B). The samples were categorized as two groups, normal skin or benign nevi (normal/benign) and melanoma. In both datasets, there was a statistically significant difference between the normal/benign and the melanoma groups. To further investigate SFRP3 expression in melanoma we used one non-transformed immortalized human melanocyte cell line, Hermes 3A [20], and three human melanoma cell lines, A375, A2058 and HTB63. We analyzed SFRP3 mRNA levels using reverse transcriptase-quantitative PCR (RT-QPCR) and SFRP3 protein levels using an ELISA kit and immunofluorescence (Figure 1C, 1D, 1E and S1). Low expression of SFRP3 mRNA was detected in the three melanoma cell lines in contrast to the higher expression in the melanocytes (Figure 1C). The same was true at the protein level; the ELISA kit detected higher SFRP3 levels in the melanocyte media than in the melanoma cell media (Figure 1D). The same pattern was also observed when the intracellular SFRP3 protein levels were analyzed and quantified by immunofluorescence (Figure 1E and S1 respectively). The fact that we detect higher SFRP3 protein levels in media from melanocytes than in media from melanoma cells indicates that SFRP3 can exert its function as a secreted Wnt modulator to a greater extent in melanocytes than in melanoma cells.

### Methylation of SFRP3 in melanoma cell lines

**Figure 2:**
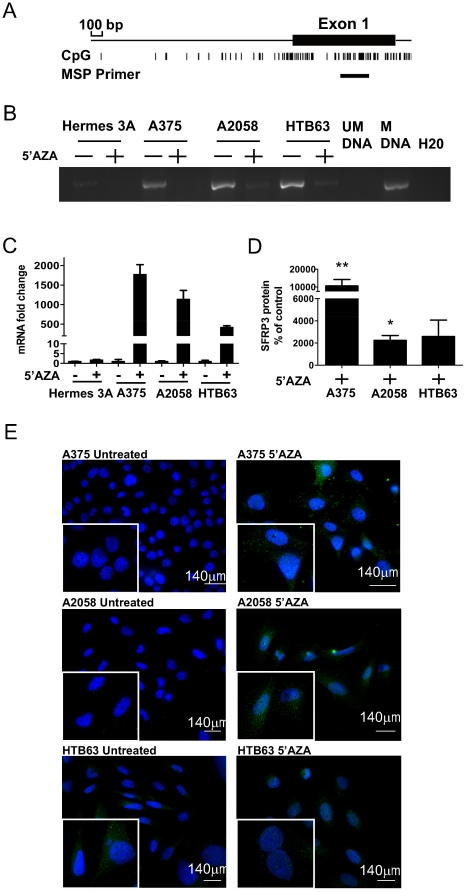
Methylation of SFRP3 in melanoma cell lines. *A*, Schematic representation of the promoter region together with the first exon of the SFRP3 gene. The CpG rich areas and the site of the methylation specific PCR primer are indicated. *B*, Methylation specific PCR. Hermes 3A, A375, A2058 and HTB63 cells were treated for 72 h with 5 µM 5′AZA and analyzed using methylation specific PCR. Universally unmethylated DNA (UM DNA) and methylated DNA (M DNA) were used as negative and positive controls, respectively. The image is representative of at least 3 independent experiments. *C*, SFRP3 mRNA expression after demethylation treatment. Hermes 3A, A375, A2058 and HTB63 cells were treated with 5′AZA as in *B* and relative mRNA expression of SFRP3 was analyzed using RT-QPCR. The graph is representative of data from 3 independent experiments and error bars denote SD within one experiment. The relative expression of each cell line is normalized to the untreated control of the same cell line. *D*, SFRP3 protein levels in the media from the indicated cell lines after demethylation treatment. A375, A2058 and HTB63 cells were treated as described for *B* and the SFRP3 protein content analyzed using the ELISA kit as described in Figure 1. The results are given as a percentage compared to non-demethylated control cells. Error bars represent SEM (n = 4). *E*, SFRP3 protein expression in the melanoma cell lines. The indicated melanoma cell lines were analyzed for their SFRP3 content by fluorescence microscopy after demethylation treatment using non-treated cells as controls. Representative immunofluorescence images from at least 3 independent experiments. * = p<0.05, ** = p<0.01.

We hypothesized that methylation might be the cause of SFRP3 down-regulation in malignant melanoma tissue and cell lines. To analyze possible methylation of the SFRP3 gene, the gene sequence and promoter region of SFRP3 [21], [22] were analyzed for CpG rich areas and one was found in the first exon (Figure 2A). This region was analyzed using methylation specific PCR in Hermes 3A melanocytes and the malignant melanoma cell lines A375, A2058 and HTB63. Methylated DNA was not present in Hermes 3A cells. However, methylated DNA was present in all three melanoma cell lines, using universally unmethylated and methylated DNA as controls, suggesting that the methylation could be a cancer specific event (Figure 2B). In melanoma cells that had been treated with 5′aza-deoxycytidine (5′AZA) there was a decrease in methylated DNA (Figure 2B). This data indicates that in malignant melanoma cells, there are methylated CpG sites in the SFRP3 gene that can be demethylated, whereas in melanocytes no methylated CpG sites could be detected. To investigate whether this methylation had a functional effect on expression of SFRP3, we used RT-QPCR and immunofluorescence. Upon treatment with 5′AZA, expression of SFRP3 mRNA did not increase in the melanocytes but there was an increase in all the melanoma cell lines (Figure 2C). We also detected increased SFRP3 protein levels in the melanoma cell lines after demethylation treatment (Figures 2D, 2E and S2). In HTB63 cells, the increase in SFRP3 protein level was detected in media but not intracellularly. These data suggest that the SFRP3 gene is methylated in malignant melanoma cells but not in melanocytes and that expression and secretion of SFRP3 can be restored in malignant melanoma cells following demethylation.

### Recombinant SFRP3 decreases migration and invasion of melanoma cells

**Figure 3:**
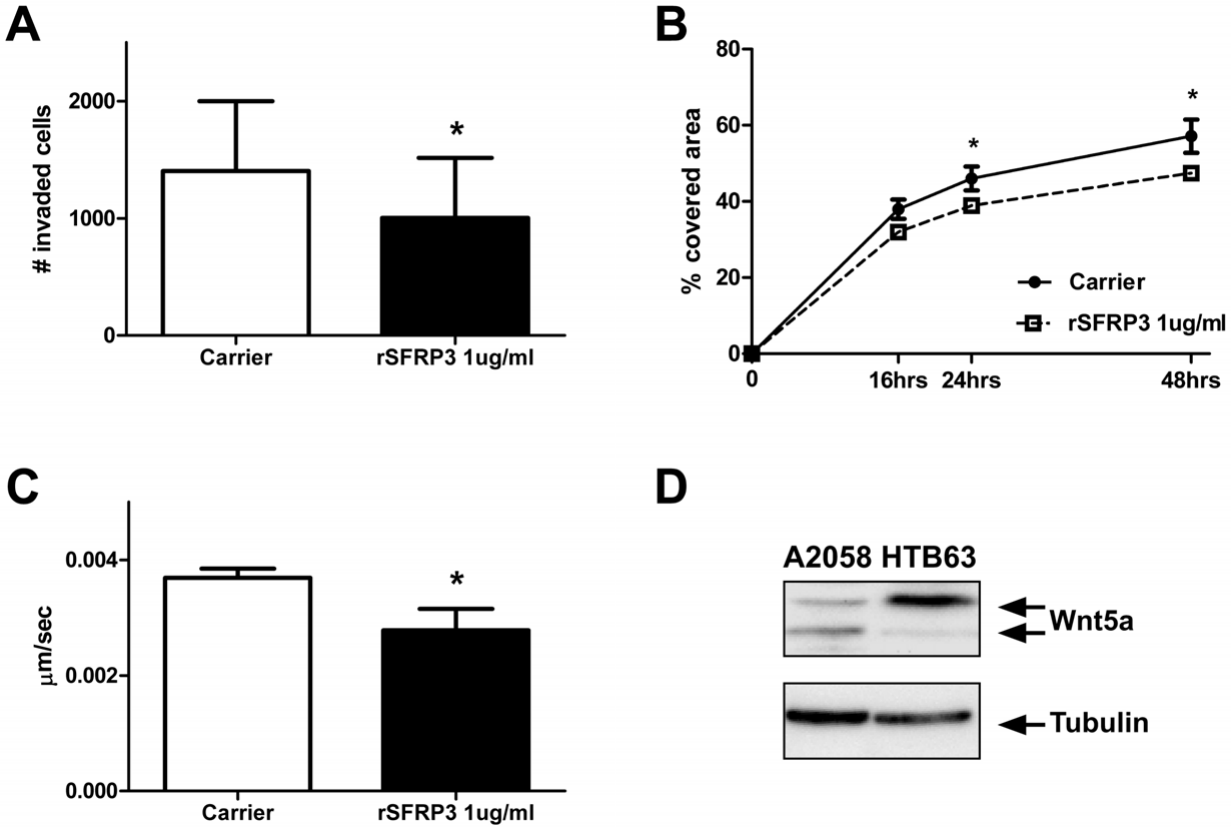
Recombinant SFRP3 decreases migration and invasion of melanoma cells. *A*, Invasion assay using Matrigel invasion chambers. A2058 cells were resuspended in serum free media and treated in the upper chamber with 1 µg/ml of rSFRP3 or carrier (0.1% BSA in PBS) and allowed to invade for 24 h. The error bars represent SEM, (n = 5). *B*, Wound healing assay. A2058 cells were treated with either 1 µg/ml of rSFRP3 (dashed line, squares) or carrier (full line, circles). Pictures were taken of the scratches at 0, 16, 24 and 48 h. Data is given as percentage of wound area closed at each time point. The error bars represent SEM, (n = 5). *C*, Migration assay, A2058 cells were treated with either 1 µg/ml of rSFRP3 or carrier in serum-free media and migration of non-dividing individual cells was analyzed manually by tracking the speed of cells for 12 h. The error bars represent SEM, (n = 5). * = p<0.05. *D*, Wnt5a protein expression analysis. Wnt5a expression was analyzed in A2058 cells and HTB63 cells using western blotting.

As Wnt signaling plays a role in melanoma progression [4], we investigated the effects of SFRP3 on proliferation/survival, migration and invasion. There was no effect of recombinant (r) SFRP3 on viable cell numbers, suggesting that SFRP3 does not affect melanoma cell proliferation or survival (Figure S3). We next investigated the effects of rSFRP3 on migration and invasion. The effect of rSFRP3 on invasion of A2058 cells was analyzed using a 3D Matrigel invasion assay. A2058 cells treated with 1 µg/ml rSFRP3 or carrier (0.1% BSA in PBS) were allowed to invade for 24 h. There was a statistically significant decrease in the number of invaded cells when treated with rSFRP3 as compared to carrier-treated cells (Figure 3A).

To investigate the effects on migration more directly, we performed wound healing assays where A2058 cells were grown to confluency and treated with carrier or rSFRP3 after which scratches were made in the monolayer. Images were taken of the scratches after 0, 16, 24 and 48 h and the scratch area was analyzed at each time point. rSFRP3 treatment significantly decreased the migration of A2058 cells (Figure 3B). To further analyze the effects of SFRP3 on melanoma cell migration, time-lapse microscopy was used. A2058 cells were treated with 1 µg/ml rSFRP3 or carrier and the speed of migration was tracked manually. Cell migration speed was decreased with rSFRP3 treatment over a 12-hour period (Figure 3C). Taken together our data show that, in malignant melanoma cells, treatment with rSFRP3 has an inhibitory effect on cell migration and invasion.

In order to investigate possible mechanisms behind the decrease in migration and invasion induced by SFRP3 in A2058 cells, we investigated the expression of Wnt5a in these cells since Wnt5a is the only Wnt protein seen to increase migration and invasion of melanoma cells [15]. We detected significantly more Wnt5a protein in HTB63 cells compared to A2058 cells, in accordance with previous findings [23]. The upper band most likely represents glycosylated Wnt5a, which is the secreted and functional form of this protein (Figure 3D). Consequently, Wnt5a is expressed in small amounts in the A2058 cells and therefore SFRP3 treatment could be assumed to have a limited effect on Wnt5a-dependent functions.

### SFRP3 inhibits migration in melanoma cells by antagonizing Wnt5a

**Figure 4:**
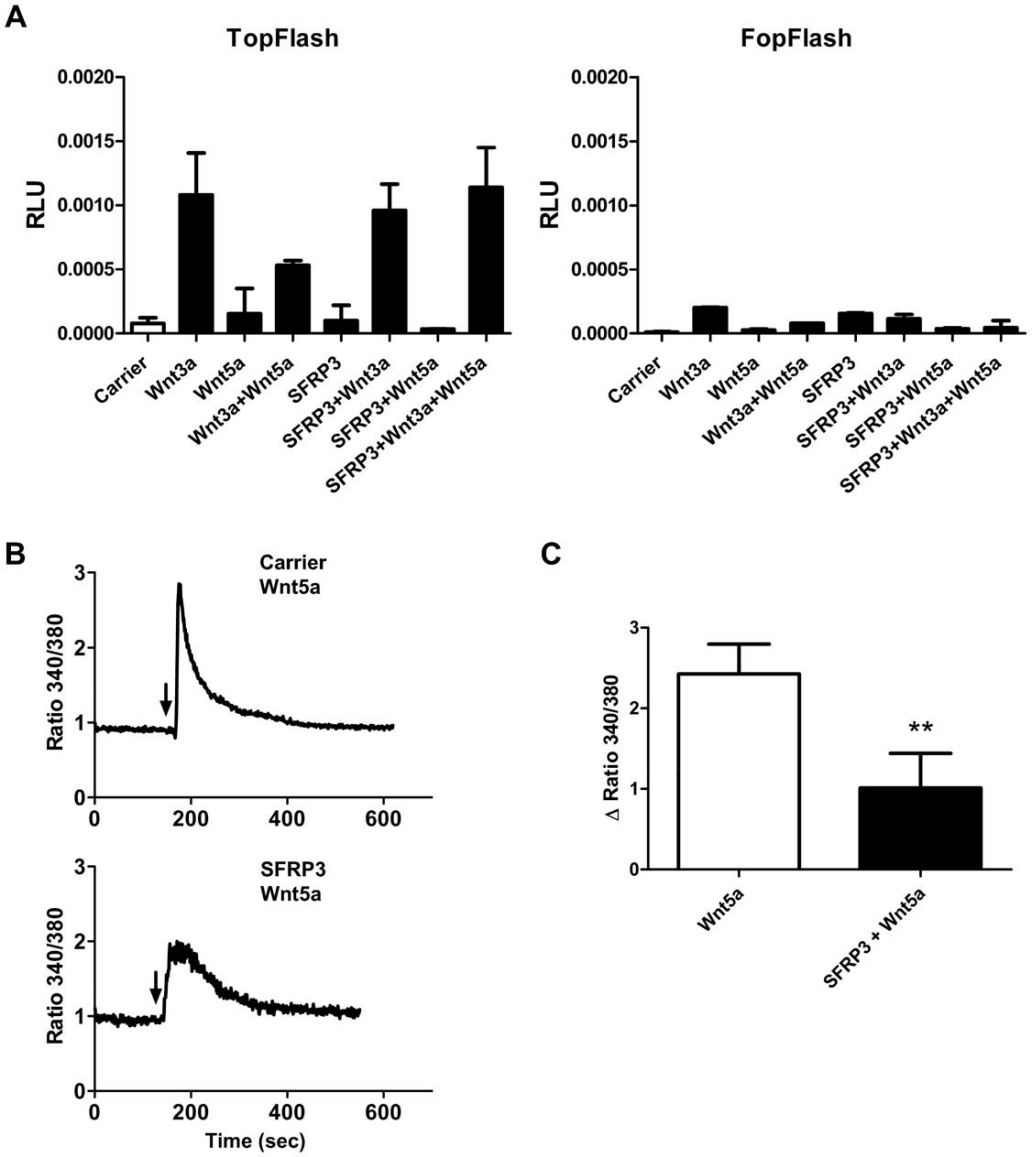
SFRP3 can inhibit Wnt5a signaling. *A*, Topflash reporter assay. A2058 cells transiently transfected with Topflash and Fopflash plasmids. Where appropriate, cells were pre-treated with 1 µg/ml rSFRP3 or carrier, and after 24 h cells were treated with the indicated combinations of Wnt ligands (0.2 µg/ml rWnt5a and 0.05 µg/ml rWnt3a) as indicated for an additional 24 h. The image is representative of 5 independent experiments and the error bars represent SD within one experiment. *B*, Measurement of intracellular Ca^2+^ signaling. rWnt5a (0.1 µg/ml, addition indicated by arrows) was added to A2058 cells pre-treated with carrier (upper panel) or 1 µg/ml rSFRP3 (lower panel). Traces are representative images of 4 separate experiments. *C*, ΔCa^2+^ ratio values from A2058 cells pre-treated with either carrier or 1 µg/ml rSFRP3 and then stimulated with 0.1 µg/ml rWnt5a. Error bars represent SD (n = 4). ** = p<0,01.

SFRP proteins have been shown to bind to and inhibit the effects of different members of the Wnt family with various outcomes depending on cellular/tissue context [24]. Since Wnt5a increases migration and invasion of melanoma cells [15] and SFRP3 and Wnt5a have previously been shown to antagonize each other [25], [26], we hypothesized that one possible mechanism behind the inhibitory effects of SFRP3 on cell migration and invasion could be inhibition of Wnt5a signaling. To investigate this, we utilized the Topflash luciferase reporter assay which measures activation of canonical Wnt signaling and its subsequent inhibition by the non-canonical signaling pathway [27], [28]. rSFRP3 did not activate β-catenin mediated transcription by itself, nor did it have an inhibitory effect on canonical signaling activated by rWnt3a, but rWnt5a could inhibit the signaling activated by rWnt3a in A2058 cells. When rSFRP3 was added to cells treated with rWnt3a and rWnt5a the inhibitory effect of Wnt5a on Wnt3a signaling was antagonized (Figure 4A).

As the Wnt/Ca^2+^ pathway has been found to be important for the invasive capacities of Wnt5a in melanoma cells [23], we analyzed the effect of SFRP3 on Wnt5a induced intracellular calcium signaling. A2058 cells were serum starved and pre-treated with carrier or rSFRP3 for 24 h before measuring intracellular Ca^2+^ signaling induced by rWnt5a. In carrier pre-treated cells Wnt5a induced a prominent increase in intracellular Ca^2+^ signaling, a signal that was inhibited by more than 50% in cells that were pre-treated with rSFRP3 (Figure 4B and 4C).

**Figure 5:**
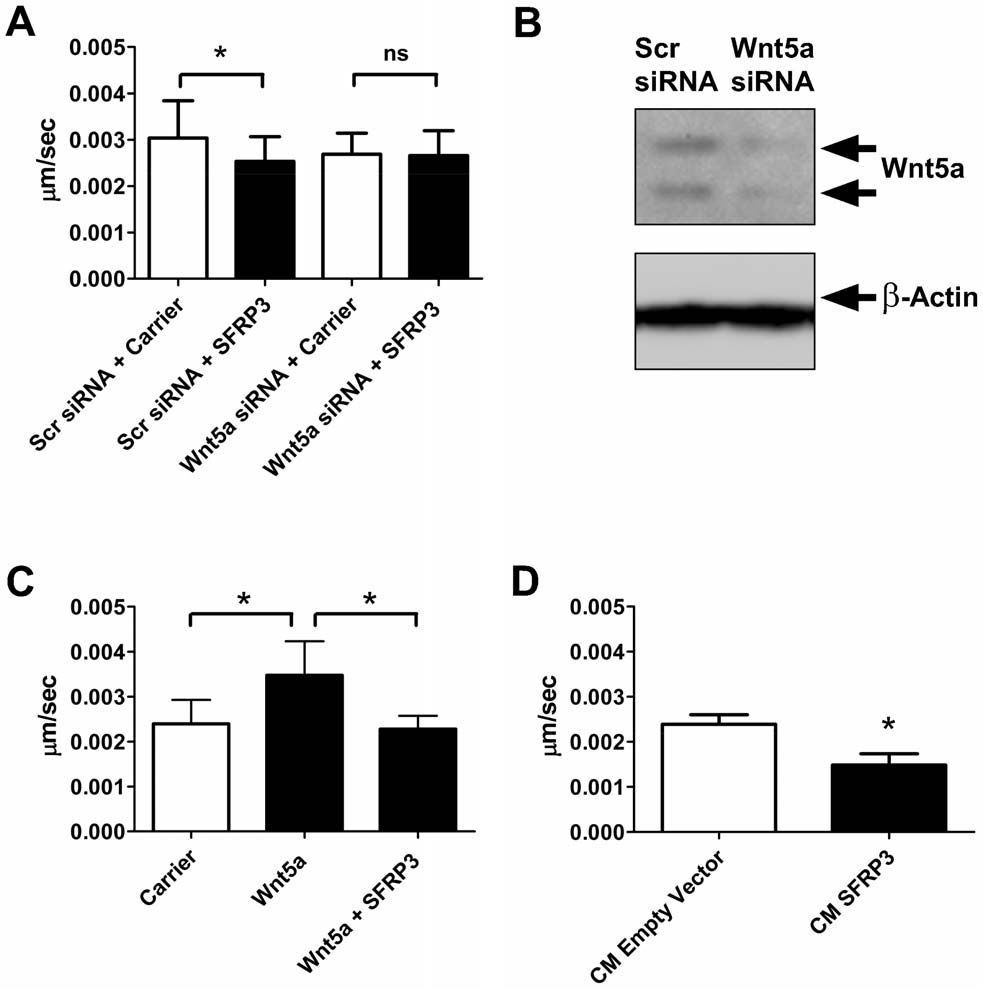
SFRP3 inhibits migration in melanoma cells by antagonizing Wnt5a. *A*, Migration assay using Wnt5a knockdown cells. siRNA transfected A2058 cells were grown in serum-free media for 24 h and then treated with 1 µg/ml rSFRP3 or carrier. Migration was analyzed using time-lapse microscopy for 24 h. The error bars represent SD, (n = 4). *B*, Wnt5a knockdown. The knockdown of Wnt5a in A2058 cells were analyzed using western blot but also by RT-PCR (Figure S4). *C*, Migration assay using rWnt5a and rSFRP3 in A2058 cells. Cells were pre-treated with either carrier or 1 µg/ml rSFRP3 in serum-free media for 24 h. The cells were then treated with rWnt5a 0.2 µg/ml and analyzed using time-lapse microscopy as in A for 24 h. The error bars represent SD, (n = 4). *D*, SFRP3 conditioned medium and migration assay. HTB63 cells were transfected with pCMV6-Empty-Vector or pCMV6-SFRP3 and kept in serum-free media for 24 h. These media were transferred to non-transfected HTB63 cells that were then analyzed using time-lapse microscopy as in A. Error bars represent SD (n = 3). * = p<0.05.

We next investigated if inhibition of Wnt5a signaling by SFRP3 was also the mechanism of the inhibition of migration observed by stimulation of melanoma cells with rSFRP3. Wnt5a expression was down-regulated in A2058 cells using siRNA. siRNA transfected cells were treated with either carrier or 1 µg/ml rSFRP3 and differences in migration were traced manually using time-lapse microscopy. In cells transfected with scrambled siRNA there was a decrease in migration upon rSFRP3 treatment. Interestingly, there was no decrease in migration after treatment with SFRP3 in cells where Wnt5a had been knocked down, suggesting that SFRP3 inhibits migration in melanoma cells via inhibition of Wnt5a (Figure 5A). The knock-down of Wnt5a was validated on the RNA and protein level by RT-QPCR and western blot, respectively (Figure S4 and 5B). In order to further validate if SFRP3 could inhibit migration induced by Wnt5a, A2058 cells were pre-treated with rSFRP3 or carrier in serum free media for 24 h after which rWnt5a was added. rWnt5a increased the migration of A2058 cells and this increase was inhibited in cells pre-treated with rSFRP3 (Figure 5C).

To complement this finding, we investigated the effects of SFRP3 in the melanoma HTB63 cells which have a higher Wnt5a expression compared to A2058 cells (Figure 3D). However, we saw no effect of 1 µg/ml of rSFRP3 on migration of these cells (data not shown), which could be due to too low a concentration of SFRP3 to have an inhibitory effect on migration of cells with a high endogenous expression level of Wnt5a. To overcome this problem we used conditioned medium from HTB63 cells transfected with a SFRP3 vector (CM-SFRP3, expression validated in Figure S5) or an empty vector (CM-EV) in order to investigate the effect of SFRP3 in HTB63 cells. In migration experiments using time-lapse microscopy there was a pronounced and statistically significant decrease in migration of cells treated with CM-SFRP3 compared to cells treated with CM-EV for 12 h (Figure 5D). The large decrease of HTB63 cell migration upon CM-SFRP3 treatment is in agreement with the high level of Wnt5a in these cells. Consequently these data are in line with the previous assumption that SFRP3 inhibits migration in melanoma cells by inhibiting Wnt5a signaling.

## Discussion

The present finding, that mRNA expression levels are lower in malignant melanoma tissue than in normal skin/benign nevi is in agreement with our observation that the SFRP3 gene is methylated in melanoma cell lines but not in the Hermes 3A melanocyte cell line. SFRP3 down-regulation has been demonstrated in other cancers [29], [30], [31], [32], [33], and it was speculated that the cause for SFRP3 down-regulation could be loss of heterozygosity in the SFRP3 loci, 2q31-33 [30], [31]. However, in parallel it was found that the other members of the SFRP family (SFRP1, -2, -4 and -5) were methylated in different types of cancer, such as breast and colorectal cancer [34], [35]. One of these studies reports no methylation of the SFRP3 gene and presumably because of this, many subsequent studies of SFRPs in cancer have not investigated SFRP3 methylation. However, McRonald *et al.* detected down-regulation of SFRP3 mRNA due to methylation in a global methylation analysis of renal cell carcinoma patient material. Furthermore, Kongkham *et al.* has detected methylation of SFRP3 in medulloblastoma, and Marsit *et al.* reported increased methylation of the SFRP3 gene in invasive bladder cancer compared to non-invasive cancer [29], [32], [33]. Taken together with previous reports on the methylation of SFRP3 in other cancers, our data suggests that gene methylation is a major reason for the down-regulation of SFRP3 in malignant melanoma.

Wnt5a signaling has been demonstrated to affect motility of melanoma whereas SFRP3 has been shown to reduce growth and motility in other types of cancer [4], [36], [37]. Therefore, we speculated that down-regulation of SFRP3 might affect the Wnt5a-driven aggressiveness of melanoma. We detected a decrease in migration and invasion in A2058 cells upon SFRP3 treatment. Since Wnt5a increases migration and invasion of melanoma cells [15], these findings supported our idea that inhibition of Wnt5a signaling could be the mechanism by which SFRP3 decreased the migration and invasion of A2058 cells. SFRP3 could inhibit Wnt5a signaling but did not induce canonical signaling nor did it inhibit canonical signaling induced by Wnt3a. SFRP3 could also inhibit Wnt5a induced intracellular Ca^2+^ signaling, a functional molecular read-out of Wnt5a signaling. In A2058 cells in which Wnt5a had been down-regulated by siRNA, the inhibitory effect on migration of SFRP3 was lost. Also, when Wnt5a was added to A2058 cells, migration was increased and SFRP3 was found to be able to inhibit this effect. In HTB63 cells that express a higher level of Wnt5a, conditioned media from SFRP3 over-expressing cells caused a large decrease in migration. Our assumption that SFRP3-mediated inhibition of Wnt5a is the mechanism by which it affects melanoma cell migration, readily explains the relatively small effect of SFRP3 on migration and invasion in A2058 cells with low expression of Wnt5a.

The inhibitory effect of SFRPs can occur either by their ability to bind Wnt receptors and induce signaling on their own or by inhibiting Wnt signaling through binding Wnt proteins or their receptors. The first option is for several reasons unlikely in the present context. First, SFRP3 alone did not induce canonical signaling in the Topflash assay. Secondly, SFRP3 had no effect by itself on migration in Wnt5a-depleted cells. We therefore conclude that the effects of SFRP3 on melanoma cell migration are due to impaired Wnt5a signaling, either via binding between the CRD of SFRPs to the CRD of the Wnt protein or its receptors, in accordance with previous findings in other systems [38], [39], [40].

The notion that SFRP3 affects melanoma cell migration via modulation of Wnt5a signaling is supported by numerous studies on how Wnt5a regulates melanoma cell migration. For example, Wnt5a can mediate migration via PKC activation, by inducing an epithelial to mesenchymal transition (EMT), by inhibiting metastasis suppressors such as matrix metalloproteases [41] and by regulating expression of tumor antigens via STAT3 [14]. Although not related to Wnt5a signaling, SFRP3 has been shown to reverse EMT, decrease activity and expression of matrix metalloproteases and decrease activation of β-catenin in other types of cancer [36], [37].

In summary, our data reveal that SFRP3 expression is down-regulated in malignant melanoma tissue and melanoma cells and can be restored in malignant melanoma cell lines following demethylation of the SFRP3 gene. Functionally, addition of SFRP3 to these cell lines decreased their migration and invasion. This effect is readily explained by SFRP3-induced inhibition of Wnt5a signaling. These data suggest that SFRP3 acts as a metastasis suppressor in malignant melanoma.

## Materials and Methods

### Cell culture

All melanoma cell lines were grown in 5% CO2 at 37°C in a humidified atmosphere. The malignant melanoma cells A375, A2058 and HTB63 were obtained from the American Type Culture Collection (ATCC Manassas, VA) and grown according to their recommendations. Hermes 3A cells were kindly provided by Professor Dorothy C Bennett, Division of Biomedical Sciences, St. George's, University of London, UK and grown according to their instructions. In brief, Hermes 3A cells were grown in 10% CO_2_ in RPMI 1640 medium supplemented with 10% fetal bovine serum, 10^5^ U/L penicillin, 100 mg/L streptomycin, 2 mM glutamine, 7.5 µg/ml phenol red, 200 nM TPA, 200 pM Cholera toxin, 10 nM Endothelin-1, and human stemcell factor, 10 ng/ml (R&D systems, Minneapolis, MN). The cells were regularly analyzed for the absence of mycoplasma contamination. All reagents were of analytical grade and purchased from Sigma-Aldrich (St Louis, MO) unless otherwise noted.

### RNA extraction, RT-PCR and QPCR

RNA was extracted according to the manufacturer's instructions using the RNeasy Plus kit (Qiagen Hilden, Germany). 1 µg of RNA was used for cDNA synthesis using random primers and M-MuLV reverse transcriptase. PCR was performed using 1× of 10×*Taq* Buffer with KCl, 1.5 mM MgCl, 0.2 mM dNTPs, 0.4 µM primers, 1 unit Taq DNA polymerase enzyme and 1–3 µl cDNA in 25 µl reactions for 30 cycles. PCR products were separated on 2% agarose gels and visualized using GelRed on a UV board. QPCR was performed with Maxima SYBR Green QPCR master mix in 25 µl reactions with 100 nM primers for 40 cycles. The housekeeping genes, UBC, SDHA and YWHAZ were used as internal controls [42] for the reactions with specific primers (all primer sequences are listed in Table 1). All PCR products were obtained from Fermentas (Burlington, Ontario, Canada).

**Table 1:** Primer sequences.

Primer sequence	Primer name
GTTGTTGTTTCGAAGGTTAGAC	Left Meth primer
AATAAAACAAAATACAACCGCG	Right Meth primer
TCAGGACCACATGCAGTA	Wnt5a F
CTCATGGCGTTCACCACC	Wnt5a R
TCCGGAAATAGGTCTTCTGTGT	SFRP 3 F
CGGAGCTGATTTTCCTATGG	SFRP 3 R
ATTTGGGTCGCGGTTCTTG	UBC F
TGCCTTGACATTCTCGATGGT	UBC R
ACTTTTGGTACATTGTGGCTTCAA	YWHAZ F
CCGCCAGGACAAACCAGTAT	YWHAZ R
TGGGAACAAGAGGGCATCTG	SDHA F
CCACCACTGCATCAAATTCATG	SDHA R

### Treatment with demethylating drugs, DNA extraction, bisulphate treatment and methylation specific PCR

Cells were treated in 10 cm plates with 5 µM 5′Aza-deoxycytidine, (5′AZA) for 72 h with medium and the drug replaced every 24 h. DNA was extracted according to the manufacturer's instructions using the DNA Blood and Tissue Kit (Qiagen). 0.5 µg of DNA was used for bisulphate treatment using the Epitect bisulphate kit (Qiagen) according to the manufacturer's instructions. Universally unmethylated and methylated DNA were used as positive and negative controls respectively for methylation specific PCR (EpiTect Control DNAs, Qiagen). Methylation specific PCR reactions were carried out as described above (all primer sequences are listed in Table 1). CpG analysis was carried out using the UCSC Genome Browser (http://genome.ucsc.edu, Feb 2009) and methylation specific PCR primers were designed using MethPrimer (http://www.urogene.org/methprimer).

### Wound healing

For wound healing experiments the cells were seeded in 35 mm plates and grown to confluency. After 24 h serum starvation, three scratches were made in each plate using a 200 µl pipette tip. Cells were then treated with carrier (0.1% BSA in PBS) or 1 µg/ml rSFRP3 (R & D Systems). Pictures of the scratches were taken at the time points indicated. Sizes of the scratches were measured using the Image-J software.

### Time-lapse microscopy

The time-lapse migration experiments were performed using cells seeded at 40–50% confluency. The cells were serum starved for 24 h and treated with either carrier (0.1% BSA in PBS), 1 µg/ml rSFRP3 or 0.2 µg/ml rWnt5a as indicated. Pictures were taken every 5–15 min over a 12 h period in a humidified 37°C chamber with 5% CO_2_. The speed of migration of non-dividing individual cells was traced manually using the Volocity software (PerkinElmer, Waltham, MA).

### Matrigel invasion assays

Matrigel invasion chambers (BD Franklin Lakes, NJ) with 8 µm pore sizes in a 24-well plate format were used. After 24 h serum starvation, A2058 cells were resuspended in serum free media and 1 µg/ml of rSFRP3 or carrier (0.1% BSA in PBS) was added to the upper chamber. 10% serum containing medium was added to the lower chamber. The cells were allowed to invade for 24 h. The membranes were wiped to remove the matrigel and fixed with 4% paraformaldehyde, stained with crystal violet and the number of cells that had invaded through to the other side of the membrane were counted.

### Antibodies, immunofluorescence and western blot

For western blotting, cells were lysed in Triton lysis buffer (50 mM Tris (pH 7,5), 1% Triton- X100, 140 mM NaCl, 0.5 mM EDTA, 0.5 mM MgCl2, 10 mM NaF, 2 mM Na3VO4, 1 mM Pefabloc and 1 µg/ml Leupeptin). 30–50 µg of protein was separated on SDS-polyacrylamide gels and transferred to PVDF membranes. Membranes were incubated with primary anti-Wnt5a antibody (1∶1000) produced as previously described [43]. α-tubulin and β-actin were used as loading controls (antibodies from Santa Cruz Biotechnology, Santa Cruz, CA). Visualization was done using Bio-Rad XRS+ and Image Lab software (BioRad Hercules, CA). For immunofluorescence cells were grown on glass slides and fixed using PBS with 5% formaldehyde, 2% sucrose. Cells were then permeabilized with 0.5% NP40, 10% sucrose and 1% calf serum and blocked for 30 min with 10% goat serum in 0.02% Tween in PBS. Cells were incubated for 1 h with SFRP3 monoclonal antibody (R&D systems 1∶100) and then with Alexafluor488 secondary antibody (1∶500) for 1 h and counterstained with DAPI (Invitrogen). The intensity of entire immunofluorescence images was measured using the Image J software and expressed as Alexa 488/DAPI ratio.

### SFRP3 ELISA

The levels of secreted SFRP3 from the different cell lines were analyzed using a commercial sandwich ELISA kit (R&D Research Minneapolis, MN). The ELISA was performed according to the manufacturer's instructions. Briefly, cells were grown in serum free media for 48 hours or for 24 hours after 5′aza treatment before the medium was removed. It was then concentrated using 10 kDa concentration columns (Millipore Billerica, MA) and subsequently analyzed using the ELISA kit. The SFRP3 levels were normalized against total protein.

### Transient transfections

For siRNA transfections, A2058 cells were transfected with 30 nM of siRNA against Wnt5a or scrambled control siRNA (Applied Biosystems, Carlsbad, CA). HTB63 cells were transfected with pCMV6-AC-GFP or pCMV6-AC-GFP-FrzB (RefSeq NM_001463, Origene, Rockville, MD) using Lipofectamine2000 (Invitrogen) transfection reagent at a ratio of 1∶2. For transfections, cells were incubated in transfection mix for 4–6 h, then kept in normal media overnight and then changed into serum-free media for 24 h. For the Topflash assay, A2058 cells were transfected with Topflash (Addgene plasmid 12456, TCF/Lef driven luciferase construct) or Fopflash plasmids (Addgene plasmid 12457, Inverted TCF/Lef driven luciferase construct) and with Renilla luciferase plasmid [44].

### Luciferase reporter assay

Transfected A2058 cells were kept in serum-free media with 1 µg/ml rSFRP3 or carrier for 24 h. After this time the cells were treated with carrier, 0.05 µg/ml rWnt3a or 0.2 µg/ml rWnt5a for 24 h in the combinations indicated in Figure 4A. Luciferase activity was analyzed according to the manufacturer's instructions using a Dual Luciferase reporter assay (Promega Madison, WI). Luciferase activity was normalized against Renilla activity. Cells were treated in duplicates and each lysate was analyzed twice.

### Measurement of calcium signaling

Determination of cytosolic free calcium levels was carried out in A2058 cells that had been pre-incubated with carrier (0.1%BSA in PBS) or 1 µg/ml rSFRP3 for 24 h before the experiment. The cells were then incubated in 4 µM fura-2/AM for 30 min, after which they were washed and placed in a chamber containing a physiologically balanced calcium medium (136 mM NaCl, 4.7 mM KCl, 1.2 mM MgSO_4_, 1.1 mM CaCl_2_, 1.2 mM KH_2_PO_4_, 5 mM NaHCO_3_, 5.5 mM glucose, 20 mM HEPES) at 37°C. Fluorescent signals of cells were recorded before and after stimulation with rWnt-5a (0.1 µg/ml) using excitation wavelengths of 340 and 380 nm and an emission wavelength of 510 nm. For each experiment, the fluorescence intensity from all cells in the field of vision was monitored, after which the calcium response of at least 10 single cells was calculated as the change in fluorescence intensity ratio (340/380 nm). The Ca^2+^ traces shown are representative of 4 separate experiments. The accumulated results of the induced Ca^2+^ signals are represented as ΔCa^2+^ (Δ Ratio 340/380 in the figures).

### Proliferation assay

2–5000 cells were seeded in 96 well plates. Cells were kept in serum-free medium for 24 h and then treated with 1 µg/ml rSFRP3 or carrier for 24 or 48 h. At the end of the experiment 10 µl of 1× WST1 reagent (Roche, Basel Switzerland) was added in 100 ul of medium. Cells were incubated for 2 h at 37°C and absorbance was then measured at 420 nm with 600 nm as reference wavelength.

### Statistical analysis

Means, standard deviations and standard error of the means were calculated and plotted using GraphPad Prism 5. In Figure 1A and B the unpaired Student's *t*-test was used, while in Figure 1D and 2D the Mann-Whitney test was used. In Figures 3 and 4 paired Student's *t*-test was used. All experiments were carried out at least three times for experiments where the Student's *t*-test was used and at least 4 times for experiments where the Mann-Whitney test was used.

## Supporting Information

Figure S1 **Fluorescence intensity measurement of basal SFRP3 protein expression in cell lines.** Melanocytes and melanoma cells were analyzed for SFRP3 expression using immunofluorescence that was quantified by measuring the intensity from at least 6 images from each of 3 separate experiments. The data are given as Arbitrary Units (AU). * = p<0.05, ** = p<0.01.
(PDF)

Figure S2 **Fluorescence intensity measurement of SFRP3 protein expression after demethylation treatment.** Melanoma cells treated with 5′aza were analyzed for SFRP3 expression using immunofluorescence that was quantified by measuring the intensity from at least 6 images from each of 3 separate experiments. The data are given as Arbitrary Units (AU). * = p<0.05.
(PDF)

Figure S3 **Analysis of rSFRP3 on melanoma cell viability.** Malignant melanoma cell lines were kept in serum free media for 24 h and then treated with 1 µg/ml rSFRP3 or carrier to investigate the difference in cell viability using a WST assay. *A*, A2058 cells treated for 24 h. *B*, A2058 cells treated for 48 h. The error bars represent SD (n = 3).
(PDF)

Figure S4 **QPCR analysis of Wnt5a knockdown by siRNA.** Wnt5a was knocked down in A2058 cells and then analyzed by QPCR. Wnt5a expression was normalized against the housekeeping genes YWHAZ, UBC and SDHA and is expressed as relative expression compared to Scrambled siRNA control.
(PDF)

Figure S5 **Analysis of SFRP3 over-expression.** HTB63 cells were transfected with pCMV6- AC-GFP or pCMV6-AC-GFP-FrzB (Origene) and kept in serum-free media for 24 h. Expression was analyzed using quantitative RT-PCR.
(PDF)
