# Supplementary figures and images for: Methylation and Loss of Secreted Frizzled-Related Protein 3 Enhances Melanoma Cell Migration and Invasion
Source: PLoS One. 2011 Apr 8;6(4):e18674. doi: 10.1371/journal.pone.0018674 (PMC3072980; doi:10.1371/journal.pone.0018674)

**Figure S1**

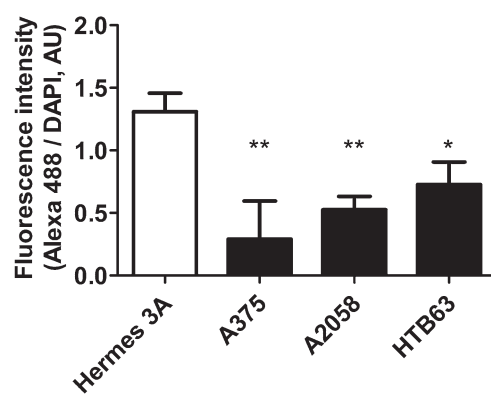

Supplement: Figure S1 — Fluorescence intensity measurement of basal SFRP3 protein expression in cell lines. Melanocytes and melanoma cells were analyzed for SFRP3 expression using immunofluorescence that was quantified by measuring the intensity from at least 6 images from each of 3 separate experiments. The data are given as Arbitrary Units (AU). * = p<0.05, ** = p<0.01. (PDF) [file pone.0018674.s001.pdf]

**Figure S2**

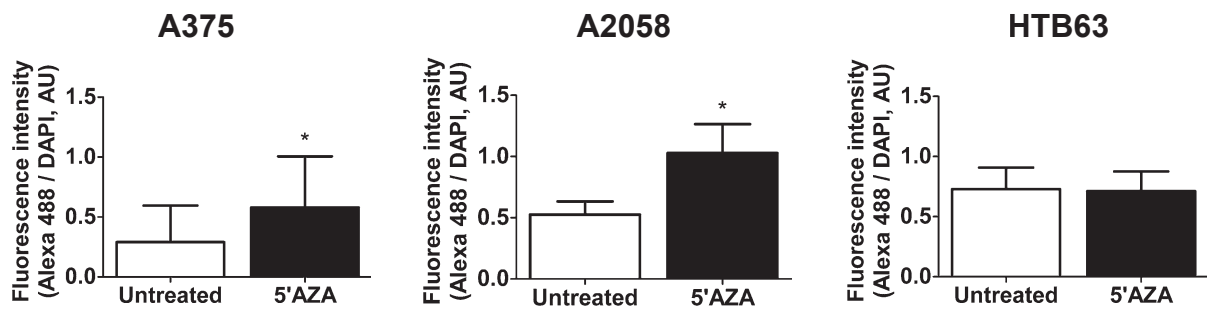

Supplement: Figure S2 — Fluorescence intensity measurement of SFRP3 protein expression after demethylation treatment. Melanoma cells treated with 5′aza were analyzed for SFRP3 expression using immunofluorescence that was quantified by measuring the intensity from at least 6 images from each of 3 separate experiments. The data are given as Arbitrary Units (AU). * = p<0.05. (PDF) [file pone.0018674.s002.pdf]

Figure S3

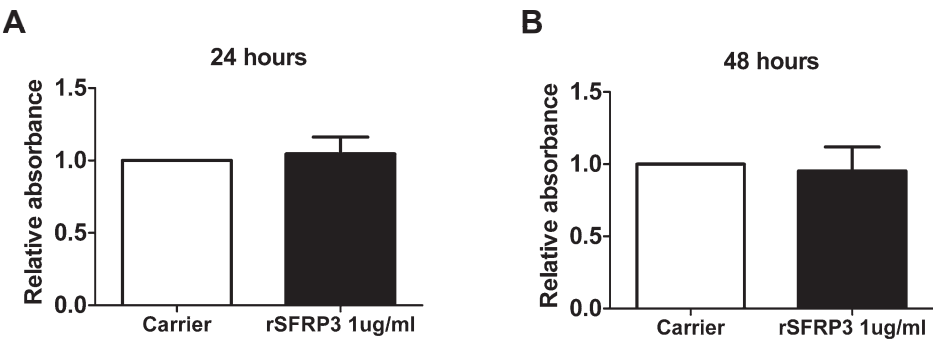

Supplement: Figure S3 — Analysis of rSFRP3 on melanoma cell viability. Malignant melanoma cell lines were kept in serum free media for 24 h and then treated with 1 µg/ml rSFRP3 or carrier to investigate the difference in cell viability using a WST assay. A, A2058 cells treated for 24 h. B, A2058 cells treated for 48 h. The error bars represent SD (n = 3). (PDF) [file pone.0018674.s003.pdf]

**Figure S4**

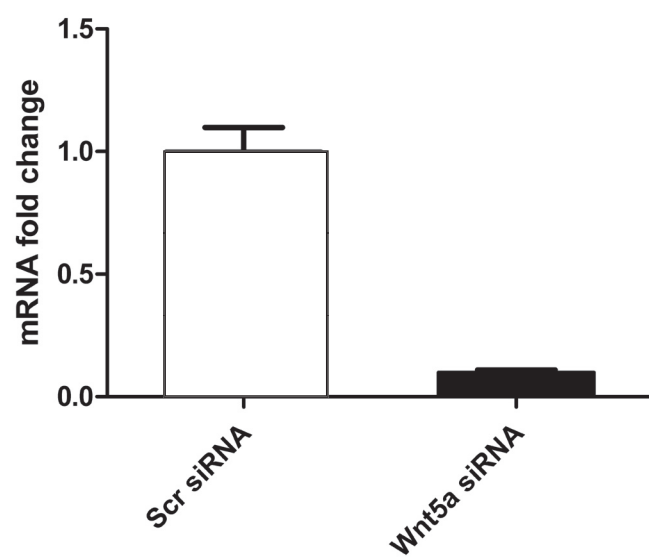

Supplement: Figure S4 — QPCR analysis of Wnt5a knockdown by siRNA. Wnt5a was knocked down in A2058 cells and then analyzed by QPCR. Wnt5a expression was normalized against the housekeeping genes YWHAZ, UBC and SDHA and is expressed as relative expression compared to Scrambled siRNA control. (PDF) [file pone.0018674.s004.pdf]

**Figure S5**

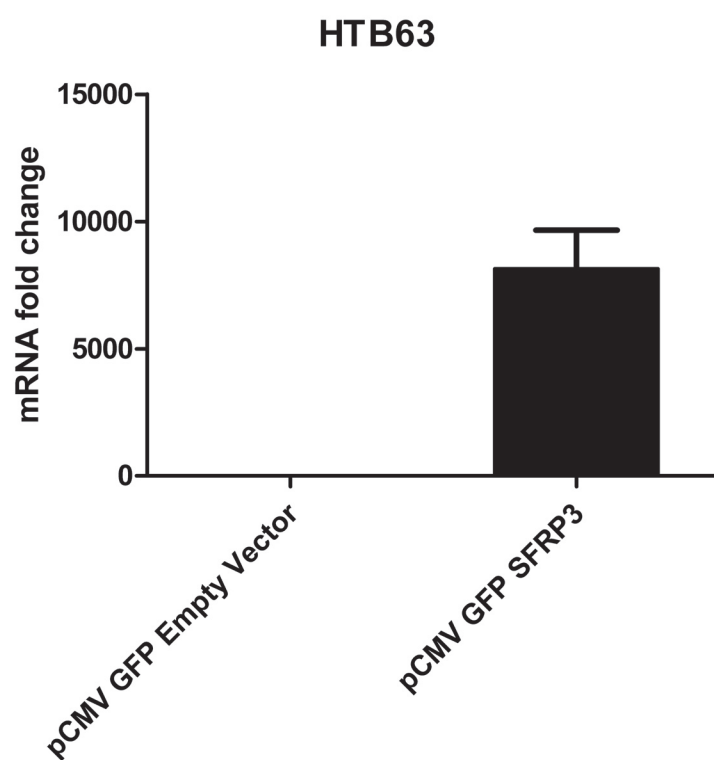

Supplement: Figure S5 — Analysis of SFRP3 over-expression. HTB63 cells were transfected with pCMV6- AC-GFP or pCMV6-AC-GFP-FrzB (Origene) and kept in serum-free media for 24 h. Expression was analyzed using quantitative RT-PCR. (PDF) [file pone.0018674.s005.pdf]
